# Multimodal transformer-based watermarking for deepfake detection and digital media authentication: current progress, challenges, and future directions

**DOI:** 10.3389/frai.2026.1848963

**Published:** 2026-07-14

**Authors:** Kok Swee Sim, Md Tahidul Islam

**Affiliations:** Department of Computer Science and Engineering, Pabna University of Science and Technology, Pabna, Bangladesh

**Keywords:** authentication, deepfake, image manipulation, multimodal transformer, watermarking, forgery detection, media forensics, cross-modal learning

## Abstract

The rapid advancement of deepfake generation technologies has fundamentally outpaced the forensic tools designed to detect and authenticate digital media. Traditional watermarking methods, while foundational, were not conceived for the adversarial complexity of multimodal content ecosystems where video, audio, and image signals are increasingly synthesized, blended, and redistributed at scale. This gap has made reliable media provenance one of the most pressing open problems in applied artificial intelligence. This mini review surveys the current landscape of transformer-based approaches to digital watermarking and deepfake detection, with a focus on their capacity to operate across multiple modalities within unified architectures. We trace the progression from classical signal-based watermarking to attention-driven deep learning frameworks, highlighting where transformer models offer meaningful resilience gains over legacy methods. We further examine emerging efforts to consolidate watermark embedding, forgery detection, and content authentication into integrated pipelines and discuss why such unification is both technically advantageous and practically necessary. The review closes by mapping the field's most consequential open challenges including cross-modal generalization, adversarial robustness, and benchmark scarcity and identifying the directions most likely to yield progress.

## 1 Introduction

The digital revolution has significantly impacted the creation, distribution, and access of media. This has opened doors to an unprecedented amount of information access. While the digital revolution has had significant positive effects, it has also had negative consequences. For instance, the recent emergence of deepfakes, which are synthetic media generated using advanced AI, has introduced an unprecedented level of risk. This is because deepfakes can mimic real faces, voices, and videos with an unprecedented level of realism. This makes it extremely difficult to distinguish between real and synthetic media (Mirsky and Lee, 2021). Digital watermarking is an essential technique for verifying the authenticity of multimedia content. This technique has been used to verify the authenticity or ownership of digital media by embedding imperceptible information within the media. Despite the significant advantages offered by digital watermarking, traditional techniques are often unable to withstand sophisticated attacks, especially those using deep learning-based techniques (Kietzmann et al., 2020).

Recent advancements in AI, especially the introduction of the transformer architecture, have significantly impacted the field of multimedia. This is because transformers, which were initially designed for natural language processing, have shown remarkable results for image, audio, and video processing (Nguyen et al., 2022). The recent success of transformers can be attributed to their ability to model complex dependencies and their capacity to integrate information from different modalities. This was made an excellent technique for robust watermarking and deepfake detection. Deep learning-based models like Wformer (Luo et al., 2024) demonstrate the importance of robust feature extraction using hybrid or attention-driven architectures. These approaches are crucial to ensure resilience against distortions and signal-level noise in multimodal watermarking and deepfake detection systems.

This mini review will cover the recent advancements at the intersection of multimodal transformers, robust watermarking, and deepfake detection. We will cover an overview of the recent advancements, challenges, opportunities, and future directions for research.

## 2 Background

### 2.1 Deepfake technology and threats

The term used for media produced through the use of advanced tools of deep learning, specifically generative adversarial networks, is called “Deepfakes,” and it may include images, audio, and video content, often indistinguishable from authentic content by Su et al. (2025). The ability to make Deepfakes has led to an increase in media fakes, and this has brought forth serious concerns related to misinformation, identity theft, and erosion of trust within society pointed by Tsai et al. (2019). Many instances have been reported, showing the potential of Deepfakes to influence societal perceptions, interfere with political processes, and compromise individual and organizational security. The recent developments related to detection are being constantly challenged by advancements related to the creation of deepfakes (Mittal et al., 2020).

### 2.2 Digital watermarking: principles and challenges

A digital watermarking method incorporates embedded information into media content without compromising the quality of the media, while also allowing users to access the embedded information when needed by Tsai et al. (2019) and Fang et al. (2025). This kind of digital watermarking is largely used for copyright protection, verification of media authenticity, and tracing any modifications made to the media content. The performance of a digital watermarking scheme is generally determined by its robustness, imperceptibility, and capacity (Wu et al., 2026). Nevertheless, traditional digital watermarking techniques face difficulties in maintaining robustness against sophisticated attacks such as those resulting from deep learning-based media manipulation and compression techniques (Tan et al., 2025; Liu et al., 2025).

### 2.3 Multimodal approaches in media authentication

In addition, it has been observed that current forms of digital media are often multimodal in nature, that is, they may contain more than one medium such as images, audio, and videos, which may be manipulated singly or in combination. Multimodal techniques use information from more than one source to improve the accuracy of authentication and detection tools (Siddharth Kashyap et al., 2026). Recent research has shown that the use of multimodal information can improve the accuracy of deep fake detection and watermarking tools (Wang et al., 2026; Nailwal et al., 2023). The use of multimodal information also poses a number of challenges such as the alignment of different features and the design of tools capable of handling different types of information (Gandhi et al., 2024).

## 3 Literature search and selection

A systematic literature search was conducted to review the recent advances in transformer-based multimodal architecture in digital watermarking and deepfake detection. Major academic databases like Google Scholar, IEEE Xplore, ACM Digital Library, Elsevier ScienceDirect, and Archive have been used in this search. Besides, some additional papers have also been included through manual screening of citation tracking and reference list. The emphasis is on publications from 2018 to 2026 (especially 2021 and beyond) to highlight the most recent advances in media authentication. A combination of keywords related to digital watermarking, deepfake detection, and multimodal deep learning have been used for the search, including “transformer watermarking,” “multimodal deepfake detection,” “vision transformer,” and “media authentication.” Studies were included if they proposed or evaluated transformer-based, attention-driven, or hybrid CNN-transformer architectures for watermarking, deepfake detection, or media authentication across image, audio, or video modalities, or contributed benchmark datasets relevant to multimodal deepfake detection. Studies were excluded if they addressed topics unrelated to digital media authentication, relied solely on traditional signal-processing watermarking techniques without any deep learning component (except where retained for historical context), lacked sufficient methodological or empirical detail, were not available in English, or represented duplicate records. Review articles were consulted separately to provide background context but were not included in the core comparative analysis. Each selected study was categorized according to its primary contribution, namely watermark embedding architecture, deepfake detection mechanism, integrated multimodal authentication framework, or benchmark dataset, allowing for structured comparison of reported robustness outcomes, performance metrics, and dataset characteristics across the reviewed literature.

## 4 Transformer models in multimedia analysis

### 4.1 Overview of transformer architectures

Transformer architectures, developed for natural language processing (NLP), have transformed the field by allowing models to understand long-range dependencies and contextual connections via self-attention mechanisms (Nguyen et al., 2022). Transformers depart from traditional RNN and CNN architectures by leveraging parallelized input processing instead of sequential computation, allowing for greater scalability and efficiency (Cui et al., 2025). At the heart of transformers is the attention mechanism, which dynamically evaluates the relevance of input components, allowing for effective modeling of intricate relationships within and between different data modalities (Qiu et al., 2024). Adversarial attacks such as Fast Gradient Sign Method (FGSM) reveal vulnerabilities in AI models, highlighting the need for robust and adaptive defense mechanisms an essential requirement for secure multimodal watermarking and deepfake detection systems (Su et al., 2025). This adaptability has enabled transformers to expand beyond NLP into fields such as computer vision and audio processing (Tsai et al., 2019).

### 4.2 Applications in image, audio, and video processing

The success of transformers in natural language processing tasks has motivated researchers to apply such models like BERT, GPT, and RoBERTa in other domains, including computer vision, audio signal processing, and video analysis. In computer vision, Vision Transformers (ViTs) have been used to obtain state-of-the-art results in image classification tasks using a sequence of patches and transformers to learn spatial relationships in images (Verdoliva, 2020). Similarly, in audio signal processing tasks, transformers have been used in speech recognition and audio classification tasks to model temporal relationships in audio signals (Li et al., 2025). Moreover, in video analysis tasks, this model is able to learn both spatial and temporal representations in videos and is used in action recognition and video captioning tasks (Shabaninia et al., 2024). In addition, some models is able to learn multiple representations and is used in audiovisual speech recognition and cross-modal retrieval task (Palanisamy et al., 2025).

## 5 Robust watermarking frameworks

### 5.1 Traditional vs. deep learning-based watermarking

Generally, conventional digital watermarking schemes have employed various signal processing techniques, including discrete cosine transform (DCT), discrete wavelet transform (DWT), and singular value decomposition (SVD), for embedding information into multimedia data (Fang et al., 2025). Single-image SNR estimation methods like QSE (Lew et al., 2025) demonstrate the importance of accurate noise modeling, which is essential for robust multimodal watermarking and deepfake detection systems. The conventional techniques are preferred for their simplicity and computational efficiency. However, they are not effective against more powerful attacks, including geometric attacks, compression attacks, and even more powerful attacks like adversarial attacks and deep learning attacks. The development of more powerful attacks against multimedia data makes it more difficult for conventional watermarking techniques to ensure robustness and imperceptibility. Recently, a number of watermarking techniques based on deep learning have been proposed, where convolutional neural networks (CNN) and even transformer architectures have been employed for improving the performance of watermarking techniques, especially against adversarial attacks and even more powerful attacks like deep learning attacks (Bertasius et al., 2021). The proposed techniques can be trained for optimizing the trade-off between robustness and imperceptibility.

### 5.2 Multimodal watermarking techniques

With the rise in multimedia data that often contains more than one modality, such as synchronized audio and video, multimodal watermarking techniques have attracted considerable research interest (Barni et al., 2001). This is because the techniques offer the possibility of improving the robustness and security of the watermarking system by utilizing the correlation between the various data modalities, thereby providing better protection against attacks that might be launched against a specific modality (Zhu et al., 2018). Current research has shown that deep learning and transformer models could be utilized in the design of multimodal frameworks that are applicable in the development of deep learning frameworks for the joint processing and authentication of various media data types (Wang et al., 2023).

## 6 Multimodal transformer-based frameworks

### 6.1 Architectural overview

Some proposed like Zhang et al. (2025) utilizes the capabilities of multimodal transformer models to ensure the embedding of strong watermarks within different media types such as images, audio, and videos. This is possible since the use of self-attention mechanisms within the multimodal transformer models enables the effective handling of relationships within and across different media types, allowing for the processing of different media streams (Khan et al., 2022; Wang et al., 2023). Most of the proposed frameworks (Fang et al., 2022) has three main modules: a multimodal encoder module that is used for feature extraction from different media types, a watermark embedding module that incorporates the watermark within the extracted media features, and a decoder module that is used for media reconstruction while maintaining the quality of the media (Fang et al., 2025).

### 6.2 Robustness and security considerations

**Figure 1:**
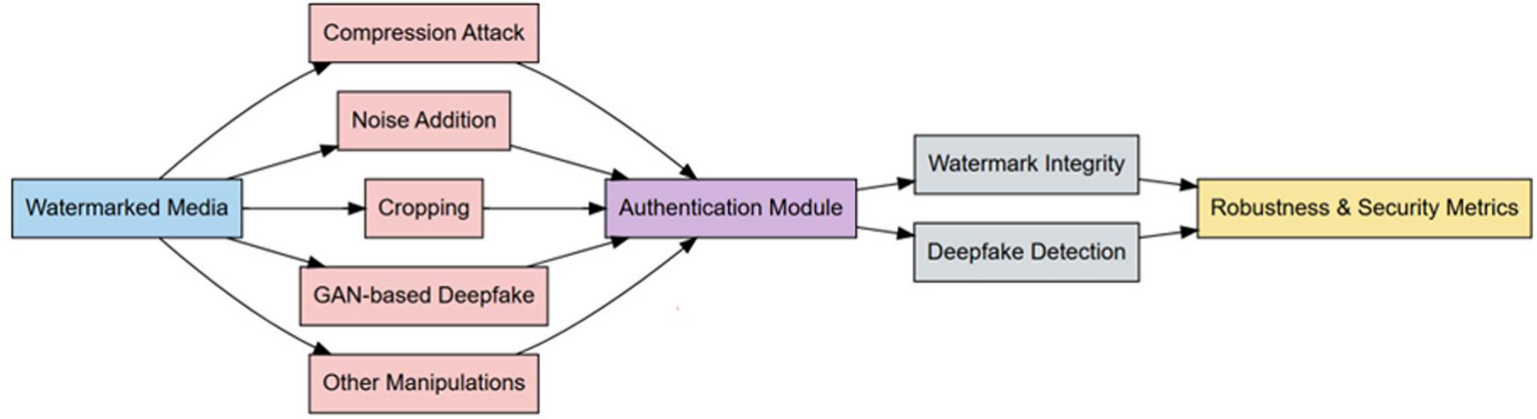
Robustness and security evaluation pipeline. Illustration of the robustness and security evaluation pipeline for the proposed framework. The watermarked media is subjected to various attacks and manipulations, including compression, noise addition, cropping, and deepfake generation. The system evaluates watermark integrity and deepfake detection accuracy under each scenario, ensuring resilience against adversarial threats.

The robustness and security of the embedded watermark need to be ensured, particularly in the presence of adversarial attacks and deepfake manipulations. The transformer-based framework has been trained on a wide variety of augmentation and adversarial attack scenarios, including compression, cropping, noise addition, and GAN-based manipulations, to improve the robustness of the watermark (Huang et al., 2025). In addition, adversarial training methods can be utilized to further improve the robustness of the model against complex adversarial attacks (Wen et al., 2025). The security of the watermark also needs to be ensured, for which cryptographic keys can be integrated into the watermarking and watermark detection processes, thus restricting access to authorized parties (Bal et al., 2018). The multimodal nature of the framework also provides redundancy, where the watermark integrity will not be threatened if an attack is carried out on one of the modalities. Table 1 summarizes various attack types and robustness outcomes for deep learning-based watermarking methods, highlighting key challenges that must be addressed in robust media protection frameworks. These findings reveal vulnerabilities to signal-level, geometric, and adversarial distortions across both image and video modalities. The overall robustness evaluation and watermark verification workflow under various attack scenarios is illustrated in Figure 1.

**Table 1:** Summary of recent deep learning and transformer-based watermarking methods and recovery: attack types, robustness outcomes, and performance metrics (2021–2026).

Method	Modality	Attack types	Robustness outcome	Key metrics	References	Remarks
WFormer (Transformer soft fusion)	Image	JPEG compression, Gaussian noise, and affine transforms	Robust	PSNR > 48 dB; high bit accuracy	Luo et al., 2024	Strong reconstruction fidelity
RoWSFormer (Swin Transformer)	Image	Rotation, scaling, and affine attacks (multi-level)	Robust	PSNR = 44.16 dB; extraction accuracy > 99%	Chen and Li, 2024	High geometric robustness
IWFormer (Transformer INN)	Image	JPEG compression, noise, and geometric distortions	Robust	NC > 0.95	He et al., 2024	Stable feature embedding
SepMark (deep separable watermarking)	Image (face)	JPEG, blur, cropping, and GAN-based deepfake manipulation	Robust	Tracer BER ≈ 0%; Detector BER ≈ 50% under deepfake	Wu et al., 2023	Dual-task watermark + detection
LampMark (training-free landmark watermark)	Image (face)	JPEG, resizing, and deepfake face swap (seven models)	Robust	~98% detection accuracy; high PSNR/SSIM	Wang et al., 2024	Training-free design
WaterVIB (variational information bottleneck)	Image	Gaussian noise, Poisson noise, JPEG, and diffusion purification	Robust	BER reduced from 3.21‰ to 0.08‰	He et al., 2026	Strong denoising resilience
MBRS (mini-batch real + simulated JPEG)	Image	JPEG Q=50, Gaussian filtering, crop, and dropout	Robust	BER < 0.01%; PSNR > 36 dB	Jia et al., 2021	JPEG-aware training strategy
SepVAMark (visual-audio fusion)	Video + Audio	Compression, noise, and deepfake manipulation	Robust	Joint source tracing + deepfake detection	Zhang et al., 2025	Multimodal watermarking
Lightweight DL video watermarking	Video	FGSM adversarial attack, compression, and noise	Partially Robust	PSNR = 38.9 dB; SSIM = 0.967; BER < 3%	Cedillo-Hernandez et al., 2026	Lightweight deployment focus
WaveVerify (audio watermarking)	Audio	MP3/AAC compression, noise, resampling, and bit-depth reduction	Robust	TPR/FPR and mIoU across 1000 samples	Pujari and Rattani, 2025	Audio authentication system

PSNR ≥ 40 dB, acceptable imperceptibility. SSIM ≥ 0.95, high visual fidelity. BER < 5% and Bit Accuracy ≥ 90% =, reliable watermark extraction. NC ≥ 0.90 = strong watermark preservation. “Partially Robust”, measurable performance drop under strongest attacks. SepMark's dual BER is intentional: Tracer BER ≈ 0% under benign distortions; Detector BER ≈ 50% under deepfake manipulation to flag tampering.

### 6.3 Deepfake detection integration

**Figure 2:**
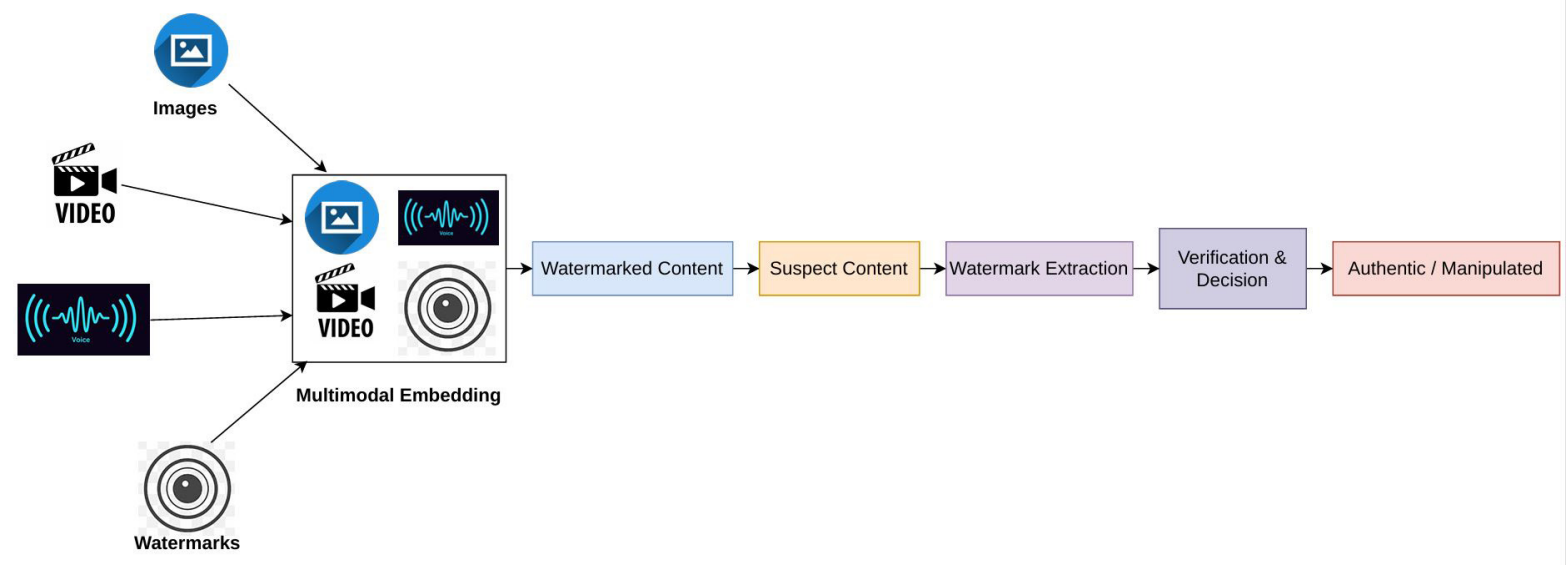
Example workflow of multimodal watermark embedding and verification. A schematic workflow illustrating the process of embedding a watermark into multimodal content (image, audio, and video) and the subsequent verification phase. The embedding module integrates the watermark into the original media, producing watermarked content. During verification, suspect content undergoes watermark extraction and authenticity checking, resulting in a decision on whether the content is authentic or manipulated.

The major advantage of some frameworks is their smooth integration with deepfake detection mechanisms. Recent methods are effective in detecting deep fake media through the joint evaluation of watermark integrity and multimodal content consistency. The transformer's ability to recognize inter-modal relationships is essential for detecting inconsistencies in deepfake media, as deepfake media creation algorithms tend to be imperfect and may not maintain perfect consistency between media types (Mittal et al., 2020; Tsai et al., 2019). Additionally, some framework can be expanded to include auxiliary deepfake detection networks for effective digital media authentication. The overall deepfake detection and watermark verification pipeline is illustrated in Figure 2.

### 6.4 Dataset availability and benchmarking

The performance and generalization capability of multimodal deepfake detection frameworks are heavily influenced by the quality and diversity of the datasets used for training and evaluation. Recent benchmark datasets encompass a broad range of manipulation techniques, modalities, languages, and real-world scenarios, enabling researchers to assess the robustness of detection models under various conditions. These datasets facilitate the evaluation of cross-modal consistency, forgery localization, explainability, and resistance to adversarial manipulations. Moreover, standardized benchmarks provide a common platform for comparing different approaches and identifying existing challenges, such as modality imbalance, limited linguistic diversity, and insufficient real-world validation. Table 2 presents a comprehensive overview of widely used multimodal deepfake datasets and benchmarks. It summarizes dataset sizes, supported modalities, key features, and benchmark specialities, while also highlighting their limitations. The table provides a comparative reference for understanding the diversity, scale, and evaluation capabilities of current datasets used in multimodal deepfake detection and forensic research.

**Table 2:** Comprehensive multimodal deepfake datasets and benchmarks.

Dataset/benchmark	Size	Modalities	Key features	Specialities	Limitations	References
ILLUSION	1.3M samples	Audio-visual	26 languages, 28 generative techniques, face swaps, audio spoofing, and synchronized A/V manipulations	Largest multimodal multilingual dataset; balanced gender/skin tone; noisy environments	Very new; limited real-world deployment validation	Thakral et al., 2025
Mega-MMDF	0.1M real + 1.1M forged (1.2M total)	Audio-visual	21 forgery pipelines (audio, visual, and face reenactment methods)	Large and diverse multimodal dataset; continuous expansion planned	Limited language diversity documentation	Zhao et al., 2025
Omni-Fake	1.2 Million+ Total Samples	Image, audio, video, and audio-video talking head	Joint detection-localization-explanation protocol	Training-ready and highly diverse, real-world social media grounding	Only supports five datasets currently	Li et al., 2026
FakeAVCeleb	20K (500 real + 19,500 fake)	Audio-video	Multimodal audio-visual manipulations	Early multimodal deepfake benchmark	Primarily English; limited language diversity	Khalid et al., 2021
WITNESS (Microsoft Benchmark)	50K+ samples	Audio-visual	Expert annotations, adversarial attack scenarios	Rigorous multimodal forensic benchmark	Evaluation-only licensing; not for training/commercial use	Roca et al., 2025
PolyGlotFake	Not specified	Multilingual multimodal	Advanced multilingual manipulation techniques	Strong multilingual and generative diversity	Dataset size not specified; very new (2024)	Hou et al., 2024
Deepfake-Eval-2024	Large-scale	Multimodal	Social media + real-world 2024 deepfakes	In-the-wild multimodal evaluation benchmark	Limited historical comparability	Chandra et al., 2026
Celeb-DF-v2	6,229 (590 real + 5,639 fake)	Video (audio-visual)	High-quality face-swapping videos	Widely used high-quality benchmark dataset	Limited audio manipulation focus	Li et al., 2019
FaceForensics++	1.8 Million+ Manipulated Images extracted from a total corpus of 4,000 fake videos	Video + Images	Automated Forgery pipeline, pixel-level localization ground truth, and multi-compression robustness evaluation	Standard benchmark for deepfake detection with four Diverse Manipulation Categories	Primarily video-based; limited audio	Rossler et al., 2019
DFDC (Deepfake detection challenge)	124K (23K real + 104K fake)	Video	8 facial manipulation algorithms; Kaggle dataset	Large-scale competition dataset	Only partial dataset publicly released	Brian Dolhansky, 2020
LAV-DF	~140K	Audio-video	Multimodal detection benchmark with structured annotations	Strong for explainable multimodal frameworks	Limited forgery method documentation	Cai et al., 2023
DeepSpeak	12K (6,226 real + 5,958 fake)	Audio-video	Lip-sync + face swap + audio manipulation	Focused on audio-driven deepfake attacks	Limited adoption; recent release (2024)	Barrington et al., 2026
ForgeryNet	221K videos (99K real + 121K fake)	Video + Image	Multiple generation methods and perturbations	Fine-grained forgery analysis benchmark	Audio modality limited	He et al., 2021
DeeperForensics-1.0	60K (50K real + 10K fake)	Video	Real-world perturbations (blur, compression, and noise)	Strong robustness testing benchmark	Limited multimodal (no audio)	Jiang et al., 2020
Wild-Deepfake	7,314 (3,805 real + 3,509 fake)	Video	Internet-collected in-the-wild deepfakes	Real-world unconstrained dataset	Noisy labels; inconsistent quality	Zi et al., 2020
DFGC 2021	Variable	Image	User-generated deepfakes (competition dataset)	DeepFake Game Competition benchmark	Primarily image-based; limited multimodal support	Peng et al., 2021
140K-Faces	140K	Image/Video	Large-scale face dataset for detection tasks	Used in explainable deepfake frameworks	Limited audio modality documentation	xhlulu, 2019

## 7 Challenges and future directions

Despite the progress made in multimodal transformer-based watermarking frameworks for deepfake detection and secure digital media authentication, there are a number of challenges that need to be addressed. Firstly, there is a challenge of scalability of transformer models that require a lot of computational resources and large datasets for effective training of the models (Tsai et al., 2019).

Secondly, there is a challenge of generalization of the watermarking and detection models. In addition, there are concerns that the models must be able to handle the changing nature of deepfakes and new forms of attacks that are being developed by attackers (Mittal et al., 2020; Tolosana et al., 2020). Furthermore, there are concerns of how to ensure the models are not affected by adversarial attacks that are meant to destroy the watermark embedded by the model (Zhang et al., 2025). Finally, there are concerns of how to effectively handle multimodal data. In addition, there are concerns of how to develop standards for evaluating the performance of the models (Singh et al., 2024). Finally, there are concerns of how to ensure that the use of watermarking and deepfake detection models does not interfere with individual freedoms and rights while providing security and meeting the needs of governments and security agencies (Dolhansky et al., 2019).

Future research should focus on improving the efficiency and scalability of transformer-based frameworks, improving the robustness of emerging threats, and developing interpretable models that provide insights into decision-making processes. Collaborative efforts between academia, industry, and policymakers will be vital to address the technical, ethical, and societal challenges associated with secure digital media authentication.

## 8 Conclusion

The rapid development in the field of deepfake technologies, along with the growing sophistication in the manipulation of digital media, poses a serious threat to the authenticity and security of digital media. The use of traditional watermarking techniques, although important, may not be sufficient to tackle the growing threat of adversarial attacks on digital media. The development in the field of transformer models, especially in multimodal settings, has opened new avenues in the development of effective watermarking techniques to detect deepfake media. In the above mini-review, the evolution in the field of digital watermarking, the revolutionary impact of deep learning models, especially transformers, and the development in multimodal settings have been discussed. The multimodal transformer model-based framework has been presented to ensure the security and authenticity of digital media. However, in spite of the development in the field of digital media security, there are still challenges to be addressed in terms of the scalability, generalization, and ethical usage of these models to ensure the security of digital media. Further research in the field is required to develop effective models to ensure the security and authenticity of digital media.
